# Heterogeneity of IGF-1 Levels in Children with hCG-Induced Precocious Puberty

**DOI:** 10.1155/2022/1068896

**Published:** 2022-11-15

**Authors:** Han Chen, Cai‐Yan Mo, Ling-Yu Zhong, Li -Yong Zhong

**Affiliations:** Department of Endocrinology, Beijing Tiantan Hospital, Capital Medical University, Beijing, China

## Abstract

**Objective:**

Sex steroid stimulates growth hormone release during puberty. However, the role of IGF-1 levels in human-chorionic-gonadotropin-induced precocious puberty remains unclear.

**Methods:**

A retrospective study reviewing thirty patients with precocious puberty due to human-chorionic-gonadotropin-secreting intracranial germ cell tumors was performed. Changes in IGF-1 levels were collected.

**Result:**

All patients included were boys. At diagnosis, the median IGF-1 standard deviation was 0.87 (0.1, 1.87). When human-chorionic-gonadotropin normalized, the median IGF-1 standard deviation was 1.58 (−0.53, 2.55), which is slightly higher than baseline (*p* = 0.408). When patients completed their therapeutic plan, the median IGF-1 standard deviation was 0.10 (−1.05, 0.68), which was significantly lower than that of baseline (*p* = 0.004) and of human-chorionic-gonadotropin being normalized (*p* = 0.003). At the last visit, the mean IGF-1 standard deviation was −1.11(−1.97, 0.76), which is slightly lower than that of baseline (*p* = 0.109) and post-therapy levels (*p* = 0.575), but significantly lower than that of human-chorionic-gonadotropin being normalized. Two patients had IGF-1 levels above 2 standard deviations at diagnosis, eight at the time when human-chorionic-gonadotropin normalized, and two at the end of therapy. Only one patient had an IGF-1 level below 2 standard deviations at diagnosis and at the time when human-chorionic-gonadotropin normalized, and two patients at the end of therapy. At the last follow-up, all patients had normal IGF-1 levels.

**Conclusion:**

IGF-1 levels in patients with human-chorionic-gonadotropin-induced precocious puberty have heterogeneity, but IGF-1 standard deviations are mostly within the normal range. If elevated, it might decline later with a decrease in human-chorionic-gonadotropin level. IGF-1 levels seem not valuable enough to assess human-chorionic-gonadotropin-induced precocity regression.

## 1. Introduction

Serum growth hormone (GH) levels increase during pubertal development as a result of increased gonadal steroid secretion. Insulin-like growth factor-1 (IGF-1) is dependent on the pulsatile secretion of GH from the anterior pituitary, which is routinely used as a marker to evaluate the activation of the GH/IGF-1 axis. Insulin-like growth factor binding protein-3 (IGFBP-3) is also a GH-dependent hormone and it correlates quantitatively with GH secretion. IGFBP-3 links to IGF-1 and its receptors in serum and then prevents them from being degraded. In the onset of puberty, both IGF-1 and IGFBP-3 spontaneously increase. In hypogonadal men, IGF-1 levels are present in a dose-dependent manner with exogenous testosterone supplementation [1]. In constitutional delay in growth and puberty, testosterone administration increases spontaneous GH secretion [2, 3] and the increment in IGF-1 levels. Furthermore, in central precocious puberty, IGF-1 levels also increase in advance, both in girls [4–14] and boys [7, 15]. Some studies prompted that elevated IGF-1 and IGFBP-3 levels commonly decrease after central puberty suppression [4, 6, 11, 15], while others considered that gonadotropin-releasing hormone agonist therapy has no significant effect on IGF-1 or IGFBP-3 levels in patients with central precocious puberty [4–8, 16, 17]. Theoretically, elevated testosterone levels would directly increase IGF-1 levels in children without the onset of gonadotropin-releasing hormone-dependent puberty. Hence, IGF-1 levels perhaps evaluate the human-chorionic-gonadotropin-induced precocity and its regression.

Intracranial germ cell tumors (iGCTs) could secrete human chorionic gonadotropin (hCG) to promote the secretion of sex steroids because their structure is similar to luteinizing hormone (LH). In hCG-induced precocious puberty, both elevated sex steroids and relevant hormones are not irreversible and they would decrease concomitantly with hCG normalized. The role of IGF-1 levels in peripheral precocious puberty remains questionable as follows. (1) Is there a transient or lasting relationship between GH/IGF-1 axis activation and peripheral precocious puberty? (2) Does the level of IGF-1 or IGFBP-3 correlate with the advancing increased sex steroid level? (3) Whether the change in IGF-1 or IGFBP-3 levels during the tumor therapy could synchronously reveal the regression of hCG-induced precocious puberty? To evaluate GH/IGF-1 activations in hCG-induced precocious puberty, this study was performed. IGF-1 and IGFBP-3 levels in patients with hCG-induced precocious puberty were analysed and compared during the therapy for hCG-secreting tumors.

## 2. Materials and Methods

Thirty boys with precocious puberty due to intracranial hCG-secreting GCTs were followed in our single center between 2019 and 2021. The diagnosis of iGCTs was made based on pathological results, tumor markers, and good response to radiotherapy or chemotherapy. The diagnosis of precocious puberty was made based on chronological age (<9 years) and premature sexual characteristics with elevated testosterone levels. The diagnostic criteria for hCG-induced precocious puberty were (1) children with elevated hCG level and low or undetectable LH or (2) children with the elevated hCG and LH levels that decreased simultaneously after chemotherapy. Tumor markers were evaluated at baseline and after every course of chemotherapy. Sex hormone profiles, IGF-1 and IGFBP-3 levels, were evaluated at baseline, the time when hCG normalized, the end of therapy, and every follow-up visit. The hCG level (range, 0–2.6 IU/L) was measured using an electrochemiluminescence immunoassay (E602, Roche Diagnostics, Basel, Switzerland). Hormones, including LH (prepubertal: <0.1–0.3 IU/L), follicle stimulating hormone (FSH) (prepubertal: male <0.1–3 IU/L), testosterone (prepubertal: <0.2–1.3 ng/dL), and IGF-1 (range, 50 ng/mL, varying with age), were measured by immunochemiluminometric assay (ICMA) (2000xpi system, SIEMENS, Washington, DC, USA). IGF-1 and IGFBP-3 were adjusted according to chronological age in Chinese children [18], expressing as a standard deviation (SD) score. The medical records were retrospectively analysed, including general characteristics, neuroimaging characteristics, laboratory examination, and therapeutic strategies.

Statistical analyses were performed using SPSS 24.0. Continuous variables with a normal distribution are presented as mean ± SD. Non-normally distributed continuous variables are presented as medians and interquartile ranges. Categorical variables are presented as a count of population. The Spearman rank correlation test was used to assess associations between initial testosterone levels and IGF-1 levels. Wilcoxon's rank sum test was used to compare IGF-1 SD and IGFBP-3 SD in different therapeutic stages.

## 3. Results

A total of 30 patients diagnosed with precocious puberty induced by iGCT and hCG were included, and all of them were boys. The mean age was 6.83 ± 1.39 years old. The median hCG level was 57.73 (32, 129) U/L in serum and 166.50 (18.48, 194) U/L in cerebrospinal fluid. The mean baseline testosterone level was 6.42 ± 3.87 ng/mL. Eleven patients presented with pineal involvement, 11 with basal ganglia involvement, 4 with sellar involvement, and 4 with bifocal lesions involving the sellar and pineal regions. All eight patients with sellar involvement but two have normal hypothalamic-pituitary-adrenal axis and hypothalamus-pituitary-thyroid axis function. Three of them had hyperprolactinemia at the beginning of the study, but was spontaneously relieved in the end of the therapy.

**Figure 1:**
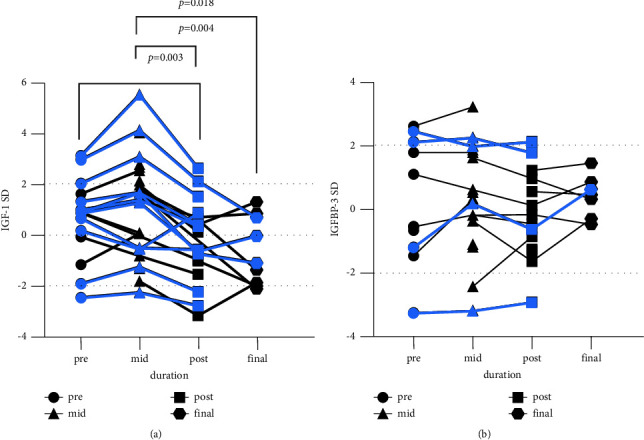
Serum IGF1 SDs/IGFBP-3 SDs in 30 boys with hCG-induced precocious puberty at diagnosis (pre), at the time when hCG level normalized (mid), at the end of multidisciplinary therapy to hCG-secreting tumors (post), and at the final follow-up visit (final). (a) IGF-1 SDs in different therapeutic phases. (b) IGFBP-3 SDs in different therapeutic phases. A total of 11 boys received IGF1 assessment and 4 boys received IGFBP-3 SD assessment (corresponding with Table 1) in the whole therapeutic process and they were marked with blue lines.

At diagnosis, the median IGF-1 SD of patients included was 0.87 (0.1, 1.87) (*n* = 18). The median IGFBP-3 SD was −0.56 (−1.2, 2.09) (*n* = 11). The correlation between the initial testosterone level and IGF-1SD is not significant (*p* = −0.318), and IGFBP-3 SD (*p* = −0.527). After the median of 2 (2, 3) courses of chemotherapy, the elevated hCG level reduced to the normal range (<2.6 IU/L). Meanwhile, testosterone levels of these boys reduced to prepuberty reference, twenty-three of them have undetectable testosterone levels, and the other seven patients' levels ranged from 0.2 to 0.68 mg/dL. At this time, the median IGF-1 SD of patients included was 1.58 (−0.53, 2.55) (*n* = 26) and the median IGFBP-3 SD was 0.28 (−0.39, 1.75) (*n* = 19). The differences in IGF-1 and IGFBP-3 between baseline and the time when hCG normalized are not significant (*p* = 0.408 and 0.910, respectively). After 7.77 ± 3.52 months of chemoradiotherapy, all patients completed the therapeutic plan. At that time, the median IGF-1 SD was 0.10 (−1.05, 0.68) (*n* = 19) and the median IGFBP-3 SD was −0.34 (−1.28, 1.00) (*n* = 14). IGF-1 SD was significantly lower after therapy compared with baseline and the time when hCG normalized (*p* = 0.004 and 0.003, respectively). The difference in IGFBP-3 SD from baseline to the end of therapy is not significant (*p* = 0.750), as well as from the time when hCG normalized to the end of therapy (*p* = 0.575). A total of 9 children received a follow-up visit with mean of 7.77 ± 3.52 months. At the last visit, they were 9 ± 1.5 years old and mean IGF-1 SD was −1.11(−1.97, 0.76), which is significantly lower than that when the hCG normalized. Furthermore, the differences in IGF-1 between the baseline level and the final follow-up visit as well as between the end of therapy and the final follow-up visit were not significant (*p* = 0.109 and *p* = 0.575, respectively). The median IGFBP-3 SD at last visit was 0.43 (−0.32, 0.83). The differences in the IGFBP-3 SD between the last visit and that in baseline, the time when hCG normalize and the end of therapy were not significant (*p* = 0.655, 0.715 and 0.173, respectively) (Figure 1).

Two patients had baseline IGF-1 levels above 2 SD, both with pineal involvement. At the time when hCG normalized, 8 patients had IGF-1 levels above 2 SD, 6 of them with pineal involvement and two with basal ganglia involvement, and meanwhile, two patients had IGFBP-3 levels higher than 2 SD with pineal lesions. At the end of therapy for the hCG-secreting tumor, two patients with pineal lesions had an IGF-1 level higher than 2 SD, and meanwhile only one patient with pineal involvement had an IGFBP-3 level higher than 2 SD. At the last follow-up, all patients had IGF-1 levels within 2 SD. Only one patient with sellar involvement had IGF-1 level lower than 2 SD at diagnosis, at the time when hCG normalized and at the end of therapy (Table 1, patient 2). Another patient with a bifocal lesion involving the sellar and pineal region also had IGF-1 levels below 2 SD at the end of therapy (Table 1, patient 5). These two patients presented with normal transaminases levels during the whole therapy.

In addition, a total of 11 patients received reevaluation of IGF-1 at diagnosis, at the time when hCG normalized, and at the end of therapy. IGF-1 levels of these patients were slightly elevated when they underwent precocious puberty, and elevated IGF-1 levels would reduce later than hCG normalized.

## 4. Discussion

Sex steroids modulate the GH/IGF-1 axis at the levels of hypothalamic GH releasing hormone secretion, pituitary GH secretion, and peripheral responsiveness to GH [19]. During pubertal onset, both the GH/IGF-1 axis and the hypothalamic-pituitary gonadal axis activate to promote the rapid growth and pubertal development. Similar to pubertal development, GH secretion presents an elevation in patients with central precocious puberty [4–15] or patients with constitutional delay in growth and puberty who were treated with exogenous testosterone [2, 3]. However, in patients with peripheral precocious puberty, whose gonadotropin-releasing hormone (GnRH) neurons have not been activated, the relationship between GH secretion and gonadal function is not clear.

The present study suggested that in patients with precocious puberty due to intracranial hCG-secreting GCTs, IGF-1 and IGFBP3 levels present heterogeneity. Most patients have the baseline IGF-1 or IGFBP-3 levels which commonly vary within the age-matched reference, not correlating with baseline testosterone levels. For partial patients with slightly elevated baseline IGF-1 and IGFBP-3 levels, these elevations would decline later than the hCG levels fell to normal range. In the end of chemoradiotherapy for tumors, IGF-1 and IGFBP-3 SDs varied but were not obviously different from the baseline. At the final follow-up visit, patients' IGF-1and IGFBP-3 levels also varied but within 2 SDs compared to their peers. Previous studies on central precocious puberty indicated that IGF-1 levels were positively correlated with LH levels [13], but not with sex steroid hormone. On the contrary, excessively high testosterone may reduce LH secretion of pituitary gland through the negative feedback regulation mechanism. It probably suggests that hCG-induced peripheral precocious puberty, rather than GnRH-dependent puberty, may not correlate with the advancing activated GH/IGF-1 axis. On the other hand, there are a lot of factors that may affect circulating IGF-1 or IGFBP-3 levels, including nutrition, body mass index, aromatase activity [3, 20], insulin, leptin level [9], and relevant metabolic parameters. Those related factors change during tumor therapy which might also contribute to the heterogeneity of IGF-1 or IGFBP-3 levels. To further explore the reason for the heterogeneity, the characteristics of patients with IGF-1 or IGFBP-3 levels beyond 2 SD were reviewed and analysed. In the present study, patients with IGF-1 or IGFBP-3 levels greater than 2 SD involved pineal gland and the basal ganglia region, conversely, those patients with IGF-1 or IGFBP-3 levels below 2 SD involved sellar region. It indicated that tumors with sellar involvement damaged anterior pituitary function. Under this situation, circulating hCG could also mimic the function of LH to promote the secretion of testosterone in tests, but growth and pubertal development were retarded. However, if the sellar lesion was metastasis in the bifocal GCTs, the effect on GH secretion will be minor and the relevant IGF-1 or IGFBP-3 level may be within the age-matched reference.

To explore the change in IGF-1 or IGFBP-3 levels during tumor therapy and its relationship with regression of hCG-induced precocious puberty, the change in hCG levels, sex hormone profiles, and IGF-1 and IGFBP-3 levels were collected and compared. These hormones were evaluated separately at baseline, the time when hCG normalized, the end of therapy, and every follow-up visit. In present study, elevated hCG levels would reduce to the normal range after the median of 2 (2, 3) courses of chemotherapy. Both variable gonadotropin and sex steroid levels would decrease with the decline of hCG levels. However, a trend towards a lower level of IGF-1 occurs after the negative of hCG. The reason for why patients may present a slightly elevated LH level is due to an immunological cross-reaction between LH and hCG. The key to differentiate GnRH-stimulated LH and hCG-induced one in these patients is to evaluate their sex hormone profiles at the time hCG became normalized. If elevated gonadotropin and sex steroid concentrations remain high when hCG normalized, the GnRH-dependent puberty should be considered, and a LH-releasing hormone test should be recommended. The delayed decline in IGF-1 is probably due to the fact that the period for IGF-1 change is longer than that for hCG and hCG-increased sex steroid levels. Unlike patients with central precocious puberty that normalizing IGF-1 levels would predict the suppressing gonadal activation [11]the change in IGF-1 or IGFBP-3 levels could not synchronously reveal the regression of hCG-induced precocious puberty.

## 5. Conclusions

In children with hCG-induced precocious puberty, IGF-1 or IGFBP-3 levels present a heterogeneity and commonly vary within an age-matched reference. The baseline IGF-1 or IGFBP-3 levels do not have the obvious correlation with the sex steroid levels. If a trend towards higher IGF-1 levels occurs, it would reduce later than the hCG level normalized. Patients with hCG-induced precocious puberty would have age-matched IGF-1 or IGFBP-3 levels after regression of precocious puberty. In conclusion, IGF-1 levels do not play a valuable enough role in monitoring and predicting the pubertal status in patients with hCG-induced precocity.

## Figures and Tables

**Table 1:** Patients with dynamic reevaluation of IGF-1 level.

No	Site	Age	Pre-hCG(U/L)	Pre-AFP(U/L)	LH (U/L)	FSH (U/L)	*T*(ng/mL)	Height SD	Pre-therapy		Mid-therapy▲		Post-therapy		Final follow-up	
									IGF-1 SD	IGFBP-3 SD	IGF-1 SD	IGFBP-3 SD	IGF-1 SD	IGFBP-3 SD	IGF-1 SD	IGFBP-3 SD
1	*P*	7	19.2	1.4	0.11	0.1	1.14	1.20	3.12	2.57	5.53	3.17	2.63			
2	*S*	7	243.5	1.45	0.2	0.1	10.3	1.11	−2.48		−2.28	−2.44	−2.80	−0.88		
3	RBG	7	112.7	2.56	0.1	0.1	7.4	1.40	0.85	−1.20	1.27	0.17	−0.65	−0.63	−0.04	0.61
4	*P*	8	50.79	293.3	0.1	0.1	6.17	0.93	0.88		1.66	−0.21	−0.75	−0.19	−1.11	−0.50
5	SP	7	152.8	2.48	0.21	0.34	9.57	1.20	−1.93	−3.25	−1.27	−3.18	−2.25	−2.92		
6	*P*	7	1430	57.33	0.12	0.48	2.1	−0.35	1.31		1.68		0.41	0.94		
7	*P*	4	378	80.85	0.21	0.29	2.33	3.82	2.03	2.43	3.08	1.96	1.51	2.10		
8	*P*	5	8.29	0.782	0.1	0.1	4.48	4.12	4.12	2.09	2.96	2.22	2.11	1.76	0.69	
9	LBG	8	73.87	1.66	0.13	1.57	8.37	0.56	0.16		−0.56	−1.20	0.87			
10	LBG	7	60	32.46	0.1	0.1	8.19	−0.07	0.98		1.39		0.34	−1.32		
11	SP	4	79.7	53.87	0.16	0.1	8.94	−0.79	0.65		−0.52		−0.58			

*P*, pineal region; *S*, sellar region; BG, basal ganglia region; hCG, human chorionic gonadotropin; AFP, alpha-fetoprotein; IGF-1, insulin-like growth factor 1; IGFBP-3, insulin-like growth factor binding protein 3; SD, standard deviation. ^▲^The time when serum hCG level reduced to normal range (0–2.6 U/L).

## Data Availability

The data used to support the findings of this study are available from the corresponding author upon request.
